# IL-9 Integrates the Host-*Candida* Cross-Talk in Vulvovaginal Candidiasis to Balance Inflammation and Tolerance

**DOI:** 10.3389/fimmu.2018.02702

**Published:** 2018-11-20

**Authors:** Giorgia Renga, Monica Borghi, Vasileios Oikonomou, Paolo Mosci, Andrea Bartoli, Jean-Christophe Renauld, Luigina Romani, Claudio Costantini

**Affiliations:** ^1^Department of Experimental Medicine, University of Perugia, Perugia, Italy; ^2^Department of Veterinary Medicine, University of Perugia, Perugia, Italy; ^3^Ludwig Institute for Cancer Research, Brussels Branch, Brussels, Belgium

**Keywords:** IL-9, mast cells, NLRP3, NLRC4 inflammasome, vulvovaginal candidiasis

## Abstract

Vulvovaginal candidiasis (VVC) is a common fungal infection caused by *Candida albicans*. The antifungal therapy represents the standard of care but due to the high costs of treatment and to the inability to prevent recurrences, the development of alternative therapeutic approaches is much-awaited. Recently, we have shown that the pathogenesis of *C. albicans* in the gut is modulated by IL-9, a pleiotropic cytokine able to promote both inflammation and tolerance during *C. albicans* infection. Herein, by using a mouse model of VVC, we similarly demonstrated that IL-9 might exert a dual role in VVC by contributing to inflammation during the initial immune activation and promoting resolution thereafter. Specifically, IL-9 has a pro-inflammatory activity at the onset of VVC by promoting NLRP3 inflammasome activity and mucosal mast cells expansion but a tolerogenic role in the resolution phase by promoting IL-1Ra production and connective tissue mast cells activation. We further show that a timely IL-9 neutralization at the onset of the inflammatory response ameliorated symptoms and vaginal pathology. Given that vaginal fluids from patients with recurrent VVC had higher levels of IL-9, these findings, by providing novel insights into the pathogenesis of VVC, may pave the way for alternative therapeutic strategies based on IL-9 neutralization.

## Introduction

The delicate balance between immune resistance and tolerance toward *Candida albicans*, an opportunistic fungus commonly present in human mucosal surfaces, is pivotal to limit inflammation and maintain homeostasis. Vulvovaginal candidiasis (VVC) is the most common mucosal fungal infection that significantly affects the quality of life, particularly in its chronic and recurrent forms (RVVC) (1, 2). The standard of care is represented by antifungal therapy. However, the high costs of treatment and the inability to prevent recurrences, currently limit the use of antifungals and make the development of alternative therapeutic approaches much-awaited (3).

IL-9 is a pleiotropic cytokine that targets different hematopoietic cells and plays a central role at mucosal sites by promoting both inflammation and protection. We have recently shown that IL-9 is able to modulate the ability of *C. albicans* to switch from commensal to pathogen in the gastrointestinal tract (4). Interestingly, a gender-specific effect seems to underlie the pathogenic role of IL-9. Indeed, we have shown that IL-9 is overproduced in female expectorates of cystic fibrosis patients and a genetic variant of IL-9 showed a sex-specific association with IgE levels in female patients (5). In addition, we have also shown that high levels of IL-9 correlates with an increased inflammation in celiac disease (4). Interestingly, celiac symptoms are not only more frequent in women than in men, but they are also more severe and quick to develop, further supporting a gender-specific effect in IL-9 pathogenicity (6). It is therefore tempting to speculate that IL-9 might play a key role in female genitourinary tract, especially in VVC.

In the present study, we evaluated the role of IL-9 in murine VVC and found that IL-9 may exert a dual role, such that a timely IL-9 neutralization ameliorated inflammation and vaginal pathology.

## Material and methods

### Mice

Female C57BL/6, 8–10 weeks for age, were purchased from Charles River. *Il9R*^−/−^ mice were kindly provided by Prof. Jean-Christophe Renauld (Ludwing Institute for Cancer Research, Brussells). Murine experiments were performed according to the Italian Approved Animal Welfare Authorization 360/2015-PR and Legislative decree 26/2014 regarding the animal license obtained by the Italian Ministry of Health lasting for 5 years (2015–2020).

### Vaginal infection

Estrogen-treated mice were inoculated intravaginally with 5 × 10^6^ viable *C. albicans* 3153A blastospores from early-stationary-phase cultures. Murine monoclonal anti-IL-9 antibody or control isotype IgG were administered intraperitoneally at the dose of 10 mg/kg starting the day of infection. Mice were sacrificed at different time points. CFUs were enumerated after incubation of Sabouraud-dextrose agar plates at 36°C for 24 h and expressed as Log CFU/100 ml of vaginal fluid. Cytospin preparations of the lavage fluids were stained with May-Grünwald-Giemsa.

### Histological and immunofluorescence staining

For histology, paraffin-embedded tissue sections (3–4 micrometer) of the vagina were stained with periodic acid-Schiff. For immunofluorescence, vaginal sections were incubated at 4°C with anti-NLRP3 and anti-pNLRC4 antibodies followed by secondary TRITC or FITC antibodies, respectively. The sections were observed using the BX51 microscope equipped with a high-resolution DP71 camera.

### RT-PCR and elisa

RT-PCR was performed using CFX96 Touch Real-Time PCR Detection System and SYBR Green chemistry (Biorad). Cells were lysed and total RNA was reverse transcribed with cDNA Synthesis Kit (BioRad), according to the manufacturer's instructions. The PCR primers sequences (5′-3′) were as follows: *Il9*, TGACCAGCTGCTTGTGTCTC and GTGGCATTGGTCAGCTGTAA; *Il1Ra*, TTGTGCCAAGTCTGGAGATG and CAGCTGACTCAAAGCTGGTG. The PCR primers for the other genes were as described (4). The levels of murine and human cytokines were determined in vaginal fluid by ELISA (R&D Systems). Data were normalized to total protein levels for each sample and expressed as pg cytokine/mg total protein.

### Statistical analysis

Student's *t*-test, one- or two-way ANOVA with Bonferroni *post-hoc* test were used to determine the statistical significance. Significance was defined as *p* < 0.05. Data are pooled results (mean ±SEM) or representative images from three experiments. GraphPad Prism software 6.01 (GraphPad Software) was used for analysis.

## Results

**Figure 1:**
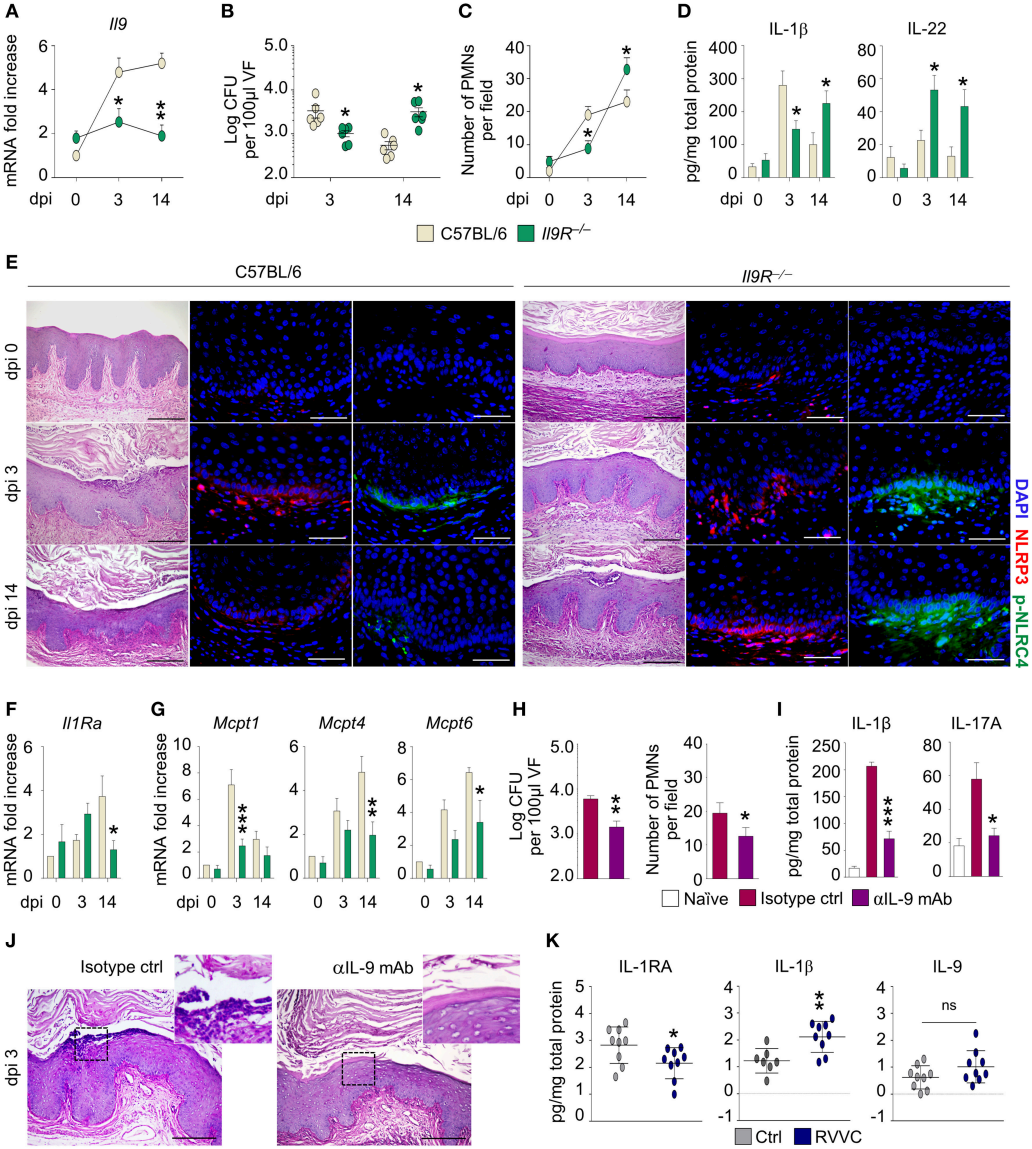
C57BL/6 and *Il9R*^−/−^ mice were intravaginally infected with *Candida* blastoconidia. **(A)** *Il9* expression (RT-PCR) in vaginal cells; **(B)** fungal burden (Log CFU/100 μl VF); **(C)** PMN recruitment in VF; **(D)** cytokine levels (ELISA) in VF; **(E)** vaginal pathology (periodic acid-Shiff-staining) and immunofluorescence staining with anti-NLRP3 or anti-pNLRC4 antibodies; **(F)** *Il1Ra* expression and **(G)** MC proteases gene expression (RT-PCR) in vaginal cells. C57BL/6 mice were infected and treated intraperitoneally with mAb neutralizing IL-9 or isotype control and assessed for **(H)** fungal burden (Log CFU/100 μl VF) and PMN recruitment in VF; **(I)** IL-1β and IL-17A production (ELISA) in VF and **(J)** vaginal pathology (periodic acid-Shiff-staining). **(K)** Cytokine production in the VF of healthy women (Ctrl) or patients with recurrent vulvovaginal candidiasis (RVVC). Data represent pooled results (mean ± SEM) or representative images from three experiments. *p < 0.05, **p < 0.01, ***p < 0.001, knockout vs. C57BL/6 mice and treated vs. untreated mice. Unpaired *t*-test, one- or two-way ANOVA, Bonferroni *post-hoc* test. dpi, days post-infection; VF, vaginal fluid; PMNs, polymorphonuclear cells; ns, not significant.

To assess the role of IL-9 in murine VVC, we resorted to C57BL/6 and *Il9R*^−/−^ mice intravaginally infected with *Candida* blastospores and first evaluated the expression of *Il9* in vaginal tissue. As shown in Figure 1A, the expression of *Il9* increased early and was observed throughout the infection in C57BL/6 mice, but was unaffected in *Il9R*^−/−^ mice, a finding suggesting that IL-9 expression is IL-9R-dependent. We next evaluated the susceptibility of *Il9R*^−/−^ mice to vaginal candidiasis. As compared to C57BL/6, *Il9R*^−/−^ mice showed a reduced inflammation in the initial phase of infection, but an increased susceptibility in a later phase, as indicated by fungal burden (Figure 1B), inflammatory cell recruitment in the vaginal fluids and mucosa (Figures 1C,E) and IL-1β production (Figure 1D). Accordingly, NLRP3, that promotes inflammation, and NLRC4, that restrains NLRP3 via IL-1Ra in VVC (7), showed a distinct pattern of activation in C57BL/6 and *Il9R*^−/−^ mice. In C57BL/6 mice, the initial activation of NLRP3 was counteracted by an increased production of IL-22 that led to NLRC4-dependent production of IL-1Ra. This resulted in blunted NLRP3 activity and return to homeostatic conditions (Figures 1D–F). Conversely, in *Il9R*^−/−^ mice, the IL-22/NLRC4 axis was not only maximally induced but also maintained throughout the infection (Figures 1D,E). Interestingly, and contrary to what expected, the high levels of IL-22 and NLRC4 were not followed by a down-regulation of NLRP3 expression in the resolution phase (Figures 1D,E). We wondered whether IL-1Ra, that mediates the suppressive effect of NLRC4 on NLRP3 activity, was impaired in *Il9R*^−/−^ mice. This turned out to be the case as *Il1Ra* was down-regulated in *Il9R*^−/−^ mice at 14 days post-infection (Figure 1F). Overall, these data suggest that IL-9 might differentially regulate NLRP3 and NLRC4 activities. Indeed, it appears to induce NLRP3 activation and IL-1β production during the onset of the inflammatory response followed by resolution of the inflammation via the NLRC4/IL-1Ra pathway. In the absence of IL-9 signaling, NLRP3 activation and IL-1β production are dampened while the IL-22/NLRC4 axis is promoted. However, the inability of the IL-22/NLRC4 axis to release IL-1Ra upon prolonged stimulation suggests that the activity of IL-9 may encompass a tight regulation of inflammasome activity. Whatever the mechanism of regulation, this finding is consistent with the observation that IL-1Ra expression becomes refractory to prolonged inducing signals (8). Overall, these data suggest that IL-9 participates in the timely regulation of inflammasome activity that takes place during VVC and that interference with IL-9 signaling might disrupt this auto-regulated circuit and promote an unrestrained inflammation.

The dual, inflammatory vs. tolerogenic, role of IL-9 in gastrointestinal candidiasis has been associated with a selective engagement of MC subsets. Indeed, IL-9 expands inflammatory mucosal MC (MMC) in the inflammatory phase, while promotes the activity of tolerogenic connective tissue MC (CTMC) in the resolution phase (4). To evaluate the role of MC in vaginal candidiasis, we assessed the expression of subset-specific MC proteases in vaginal tissue. In line with previous data, we observed that in C57BL/6 mice the increased expression of the MMC-related chymase, *Mcpt1*, occurred early while the expression of the CTMC-related chymase, *Mcpt4*, and tryptase, *Mcpt6*, occurred later (Figure 1G). As expected, the expression of MMC- and CTMC-specific proteases was impaired in *Il9R*^−/−^ mice in the initial and late phase, respectively (Figure 1G). Given that IL-9 contributes to inflammation in the early phase of vaginal candidiasis, we asked whether a timely administration of an IL-9 neutralizing antibody could ameliorate vaginal pathogenesis. For this reason, we infected C57BL/6 mice with *C. albicans* and concomitantly administered either an anti-IL-9 neutralizing antibody or isotype control. As expected, the neutralization of IL-9 clearly reduced fungal burden (Figure 1H) and restrained inflammation after 3 days of infection as showed by decreased inflammatory cytokine production and neutrophils recruitment at the vaginal parenchyma in absence of epithelial damage (Figures 1I,J).

Finally, we assessed the translatability of the results obtained in the murine model to human VVC. In agreement with previous data, the vaginal fluids of patients with recurrent VVC (RVVC) had lower levels of IL-1RA and higher amounts of IL-1β than healthy controls (Figure 1K). In addition, we observed increased levels of IL-9 (Figure 1K). Although the difference did not reach statistical significance, likely because of the low sample size, this result supports the hypothesis that IL-9 might contribute to inflammation also in human VVC.

## Discussion

The present study sheds light on a possible pathogenic mechanism in VVC involving IL-9 as a key player of inflammation but also tolerance in response to the fungus. This dual role of IL-9 in VVC parallels our previous study in the gastrointestinal tract in which we have shown that IL-9 can modulate *C. albicans* commensalism and pathogenicity (4). Collectively, our results support a model in which MC and IL-9 participation in the host-*Candida* cross-talk is not tissue-restricted but likely involved in the general mucosal response to *Candida* colonization. For instance, *Candida* induces IL-9 in a skin-tropic T cell population (9), suggesting that IL-9 might be a key player also in cutaneous candidiasis with a possible involvement of MC.

This study shows that, in the initial phase of VVC, IL-9 has a pro-inflammatory activity by promoting NLRP3 inflammasome activity and MMC expansion. Accordingly, the IL-9 neutralization early in infection ameliorates inflammation and restores epithelial homeostasis suggesting a potential therapeutic use of IL-9 neutralizing antibody in the treatment of *Candida* infection. On this regard, MEDI−528, a humanized mAb against IL-9, is already used in clinics for the treatment of asthma (10). Should the results obtained in the mouse model be confirmed in human RVVC, the use of this antibody could represent a valid therapeutic option with a quick and straightforward bench-to-bedside transition, at least in the acute symptomatic candidiasis. Herein, we show that vaginal fluids from RVVC patients have lower concentrations of IL-1RA and higher levels of IL-1β as compared to healthy control, which is consistent with our findings in animal studies. We also show that IL-9 levels tend to increase in RVVC subjects compared to healthy subjects. Although the difference was not significant, likely due to the low sample size, nevertheless it is tempting to speculate that a similar pathogenic mechanism is also effective in humans.

Quite interestingly, the role of IL-9 in VVC would also be consistent with the observation that a gender-specific effect underlies the pathogenicity of IL-9. Indeed, in patients with diseases as different as cystic fibrosis and celiac disease, the severity is higher in females than males (5, 6). It is tempting to speculate that sex hormones, whose modulatory activity of the host immune response to pathogens is well-established (11), potentially influence IL-9 expression as already shown for other cytokines (12). In addition, we have previously shown that a non-synonymous IL-9 polymorphism (rs2069885), known to be associated with lung function and sensitization, correlates with high IgE levels in female cystic fibrosis patients (5). It is tempting to speculate that IL-9 SNPs might not only influence the risk of *Aspergillus* allergy, especially in females, but could also impact the risk of *Candida* infection.

In conclusion, our study suggest the IL-9 and MC are involved in the cross-talk between the host and *Candida* in vaginal mucosa and an unbalanced response might lead to an inflammatory condition. The elucidation of molecular mechanisms underlying the interaction within the triad IL-9-MC-*Candida* may lead to the development of novel therapeutic approaches leading to the control of the inflammatory response in VVC.

## Ethics statement

Statement involving human subjects: This study was carried out in accordance with the recommendations of the University Ethics Committee of Perugia (Prot. 2012-028) with written informed consent from all subjects. All subjects gave written informed consent in accordance with the Declaration of Helsinki. The protocol was approved by the University Ethics Committee of Perugia (Prot. 2012-028).

## Author contributions

GR and VO designed the experiments. MB performed immunofluorescence and most of the *in vivo* experiments. J-CR provided the knockout mice. PM, AB, LR, CC, and GR analyzed the data and wrote the paper.

### Conflict of interest statement

The authors declare that the research was conducted in the absence of any commercial or financial relationships that could be construed as a potential conflict of interest.

## Abbreviations

VVC: vulvovaginal candidiasis
RVVC: recurrent vulvovaginal candidiasis
NLRP3: NLR family Pyrin domain containing 3
NLRC4: NLR family CARD domain-containing protein 4
MC: mast cells
MMC: mucosal mast cells
CTMC: connective tissue mast cells.
